# Supplementary material for: The interactome of intact mitochondria by cross-linking mass spectrometry provides evidence for coexisting respiratory supercomplexes
Source: Mol Cell Proteomics. 2017 Dec 8;17(2):216–32. doi: 10.1074/mcp.RA117.000470 (PMC5795388; doi:10.1074/mcp.RA117.000470)
Supplement: Supplemental Data [file supp_RA117.000470_133922_0_supp_23978_fzffwf.zip › spectra_annotation/mito_DR_spectra_annotation/142-1-2-1-1-1.pdf]

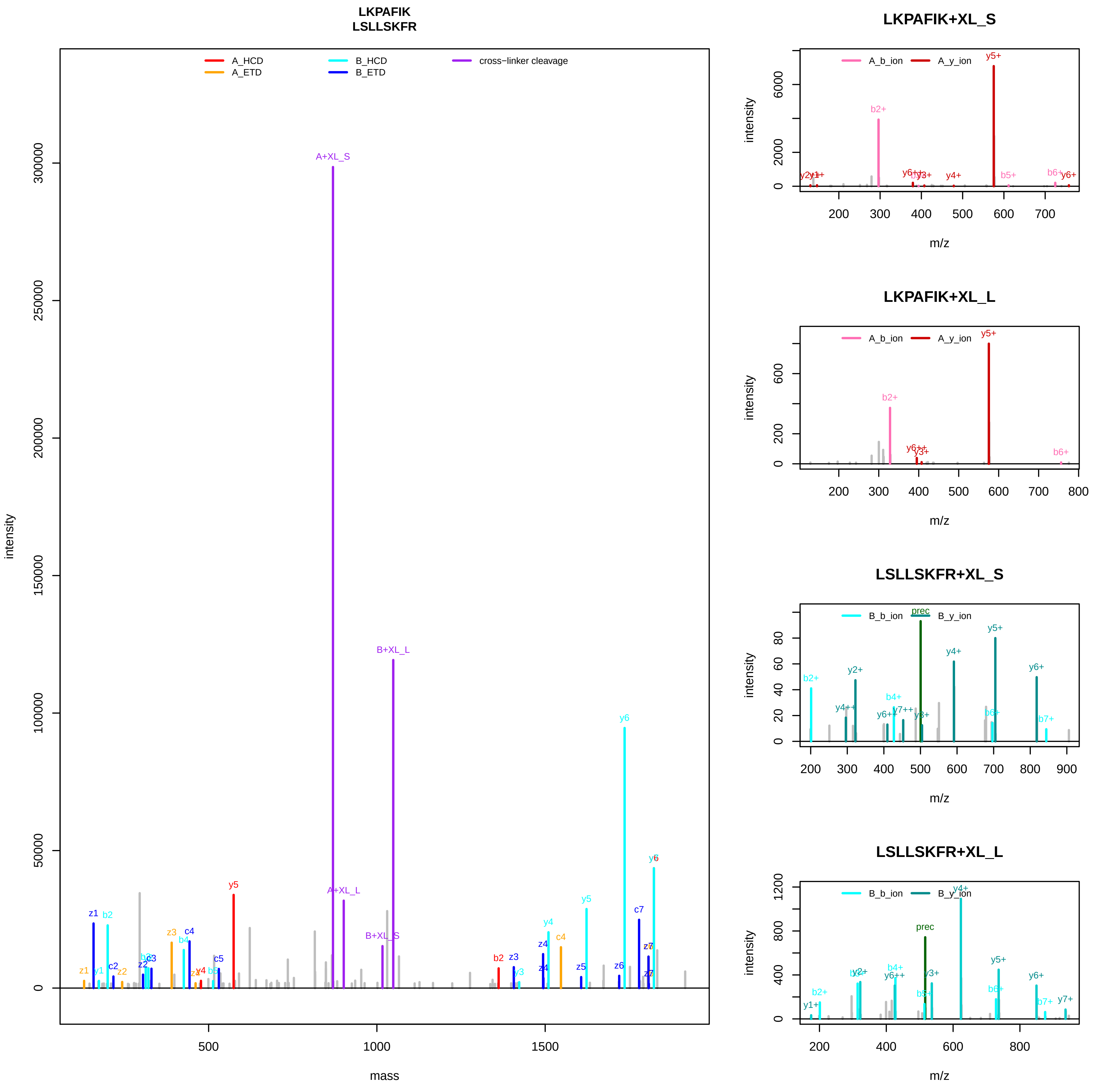

LKPAFIK
LSLLSKFR
A_HCD
A_ETD
B_HCD
B_ETD
cross−linker cleavage
intensity
mass
A+XL_S
B+XL_L
A+XL_L
B+XL_S
y6
y5
y4
c7
z7
z6
z5
z4
z3
b2
c4
y3
c5
b5
y4
z3
b4
c4
z2
c3
c2
z2
z1
b2
y1
LKPAFIK+XL_S
LKPAFIK+XL_L
LSLLSKFR+XL_S
LSLLSKFR+XL_L
A_b_ion
A_y_ion
B_b_ion
B_y_ion
m/z
prec
y5+
b2+
y4+
y6+
b6+
b5+
y3+
y2+
b4+
b7+
y1+
